# Hernia

**Published:** 1887-09

**Authors:** W. W. Walker

**Affiliations:** Schulenberg, Texas


### HERNIA.

*By W. W Walker, M. D., Schulenberg, Texas.*

For Daniel's Texas Medical Journal.

DURING the seventeen years I have practiced medicine in Fayette county, I have treated about sixty cases of strangulated hernia.

I have yet to meet a case of strangulated inguinal hernia that could not be reduced by the following method: Give a hypodermatic injection of morphia and atropia as near the constriction as possible; place a rubber bag or towel of pounded ice over the protrusion, allow it to remain ten minutes; administer chloroform to complete anæsthesia, turn a common chair down in the bed, lift the patient on its incline, which will place his body at an angle of 35 or 40 degrees. Many cases will be spontaneously reduced by intestinal gravitation, or can be, by assistance of gentle taxis. I have had eight cases of strangulated femoral hernia; six were reduced by the above method, two were operated upon.

Case 1. Mrs. Mary Meucek, aged 46 years, mother of several children, occupation Bohemian midwife; patient of Dr. G. S. Holman, who requested me to assist him. The bowel had been incarcerated thirty hours; operation made at 10 a. m., May 15, 1886. Very little serum was found in the sac; after nicking the constriction upwards and inwards, the small intestine was easily replaced, the wound closed with silver wire sutures, a dressing of carbolized absorbent cotton placed over it, and a rubber bandage applied. The surroundings were filthy,—the outlook gloomy; however, owing to the care and good attention of Dr. Holman, the case was discharged, permanently cured, June 5th.

Case 2. Sister D., aged 36 years; German; occupation, teacher. Had right femoral hernia for two years; wore a truss. Wednesday, July 20, 1887, she had a chill and fever, and during an attack of vomiting the gut was forced down, and couid not be returned. I was called Saturday morning, and made an effort to reduce it under chloroform, but soon found I could accomplish nothing. Returned to Schulenburg to procure assistance and instruments, Dr. F. A. Schmitt kindly accompanying and assisting me. He tried for some time to reduce by his method of taxis, as published in January, 1885, but succeeded no better than I had.

Operation was made at 11 a. m., July 23. When the sac was opened, at least a pint of bloody serum was discharged. Upon examination, we found about four inches of the colon tightly constricted, very dark colored, with numerous spots nearly in a state of gangrene. There was considerable trouble in returning the bowel, and the constriction had to be cut in half-a-dozen places before we could return the gut. The wound was closed with silk sutures, dusted with iodoform, covered with a pad of absorbent cotton, and a rubber bandage applied. Prognosis was very grave on account of the condition of the bowel, and the chills and fever. She was given ice and iced milk, and five grains quinia, and twenty drops tinct. ferri per chlor. every six hours. Twenty-two hours after operation, her temperature rose to 101, it being her chill day, but never again went over 99; patient was discharged, cured, August 14.

Owing to the frequency and danger attending hernia, any method promising an advance in our treatment should be hailed with delight. Dr. A. C. Bernays, in an article in the *St. Louis Medical and Surgical Journal*, for May, 1887, recommends a subcutaneous division of Pouparts' ligament, which will relax the constriction, and permit reduction,—and gives two cases successfully treated by him. I am so well satisfied with the correctness of Dr. Bernay's theory, that I shall give it a trial in the future, before resorting to the graver operations; and would respectfully ask my professional brethren to give it a trial, and report results.

I am a strong advocate of Heaton's method for the radical cure of hernia, as it is practiced by Dr. Warren, of Boston, and DeGarmo, of New York. Warren's instrument is complicated and costly. I use DeGarmo's, which is cheap, and answers every purpose. All small hernias of recent date can be cured by this method. My success has been seventy-five per cent., taking all the cases I could get. I have not attempted to present anything new, but to give a short synopsis of my work in this field.

